# Bone Defect Treatment in Regenerative Medicine: Exploring Natural and Synthetic Bone Substitutes

**DOI:** 10.3390/ijms26073085

**Published:** 2025-03-27

**Authors:** Angelo Santoro, Andrea Voto, Luigi Fortino, Raffaella Guida, Carolina Laudisio, Mariarosaria Cillo, Anna Maria D’Ursi

**Affiliations:** 1 Department of Pharmacy, University of Salerno, 84084 Fisciano, Italy; asantoro@unisa.it; 2 Scuola di Specializzazione in Farmacia Ospedaliera, Department of Pharmacy, University of Salerno, 84084 Fisciano, Italy; l.fortino4@studenti.unisa.it; 3 Department of Orthopaedics and Traumatology, AORN “San Giuseppe Moscati”, 83100 Avellino, Italy; andrea.voto.av@gmail.com; 4 Presidio Ospedaliero “Villa Malta” di Sarno, Azienda Sanitaria Locale di Salerno, 84087 Sarno, Italy; rf.guida@aslsalerno.it (R.G.); c.laudisio@aslsalerno.it (C.L.); 5 Dipartimento Farmaceutico, Azienda Sanitaria Locale di Salerno, 84124 Salerno, Italy; m.cillo@aslsalerno.it

**Keywords:** bone regeneration, fracture healing, tissue engineering, bone substitutes, scaffold biomaterials

## Abstract

In recent years, the management of bone defects in regenerative medicine and orthopedic surgery has been the subject of extensive research efforts. The complexity of fractures and bone loss arising from trauma, degenerative conditions, or congenital disorders necessitates innovative therapeutic strategies to promote effective healing. Although bone tissue exhibits an intrinsic regenerative capacity, extensive fractures and critical-sized defects can severely compromise this process, often requiring bone grafts or substitutes. Tissue engineering approaches within regenerative medicine have introduced novel possibilities for addressing nonunions and challenging bone defects refractory to conventional treatment methods. Key components in this field include stem cells, bioactive growth factors, and biocompatible scaffolds, with a strong focus on advancements in bone substitute materials. Both natural and synthetic substitutes present distinct characteristics and applications. Natural grafts—comprising autologous, allogeneic, and xenogeneic materials—offer biological advantages, while synthetic alternatives, including biodegradable and non-biodegradable biomaterials, provide structural versatility and reduced immunogenicity. This review provides a comprehensive analysis of the diverse bone grafting alternatives utilized in orthopedic surgery, emphasizing recent advancements and persistent challenges. By exploring both natural and synthetic bone substitutes, this work offers an in-depth examination of cutting-edge solutions, fostering further research and innovation in the treatment of complex bone defects.

## 1. Introduction

In regenerative medicine and orthopedic surgery, treating bone defects resulting from trauma, degenerative diseases, or congenital conditions has led to intensified research efforts to develop innovative and effective therapeutic strategies. Bone tissue is the human body’s structural framework, supporting mechanical loads and facilitating movement through muscle attachment points. Despite its intrinsic regenerative capacity, complex fractures and critical-sized bone defects can significantly impair the healing process, often leading to nonunions. Bone healing occurs through two primary mechanisms: direct healing, which requires precise fracture reduction and stabilization without callus formation, and indirect healing, characterized by callus-mediated bone regeneration, the latter being the predominant physiological response. The repair process begins with the inflammatory phase, which is triggered immediately after injury. This phase, lasting several days, is marked by hemorrhage due to vascular disruption within the bone marrow, cortical bone, and periosteum, leading to hematoma formation. Platelet aggregation initiates the release of pro-inflammatory cytokines, including tumor necrosis factor (TNF)-α and interleukin (IL)-1 and IL-6. At the same time, neutrophils and macrophages infiltrate the injury site to mediate tissue repair [1,2]. Concurrently, mesenchymal stem cells (MSCs) derived from the bone marrow migrate to the damaged area and differentiate into osteoblasts and chondrocytes [3,4]. Several studies suggest that cytokines such as bone morphogenetic proteins (BMP-2 and BMP-7) play a pivotal role in MSC differentiation [5,6,7]. Vascular disruption at the injury site induces localized hypoxia, stimulating the expression of hypoxia-inducible factor (HIF), which in turn upregulates vascular endothelial growth factor (VEGF), thereby promoting angiogenesis and facilitating tissue repair [8]. The reparative phase follows in the subsequent weeks, during which bone fragments are bridged by a soft callus primarily composed of fibrous tissue, with progressive calcium deposition within the osteoid matrix. Further ossification leads to the formation of the hard callus [9]. Although bones appear hypertrophic due to the accumulation of callus tissue during this phase, their mechanical integrity remains suboptimal [10]. The final remodeling phase extends over several months to years, during which angiopoietin- and VEGF-mediated pathways drive the replacement of woven bone with structurally superior lamellar bone [11,12,13]. Bone remodeling is a continuous, dynamic process characterized by cycles of osteoclastic resorption and osteoblastic formation, ultimately restoring the original bone architecture through callus resorption and enhanced mineralization [14,15]. Regenerative medicine, particularly tissue engineering-based approaches, presents promising solutions for the treatment of nonunions and extensive bone defects that remain challenging to manage with conventional therapies [16,17,18]. Three principal components are involved: (i) cellular elements, (ii) bioactive growth factors, and (iii) biomaterial scaffolds [19]. Regarding cellular therapies, three primary types of stem cells are employed: somatic stem cells (naturally occurring within the body), embryonic stem cells (derived from cultured embryos), and induced pluripotent stem cells (reprogrammed from differentiated cells) [20,21,22,23]. Among these, somatic stem cells, particularly MSCs, have demonstrated substantial potential in regenerative applications due to their ability to differentiate into multiple musculoskeletal tissues, including bone, cartilage, and adipose tissue [24,25,26]. Growth factors play a crucial role in bone regeneration by stimulating cellular proliferation and differentiation. Following a fracture, these biomolecules are secreted in the microenvironment surrounding the injured tissue, orchestrating the recruitment and activation of bone marrow-derived MSCs [27,28]. However, spontaneous regeneration may be impaired when bone defects exceed a critical threshold or endogenous growth factor levels are insufficient [29]. To overcome these limitations, the synergistic application of bioactive scaffolds and exogenous growth factors has been explored [30,31]. Autologous cancellous bone grafts currently represent the gold standard in bone replacement therapy due to their osteogenic, osteoconductive, and osteoinductive properties [32,33]. Despite their widespread use, bone grafts are associated with inherent risks and clinical limitations. The need for additional surgical procedures to harvest autologous grafts increases hospitalization duration and healthcare costs. Moreover, donor site morbidity, including structural and aesthetic complications, represents a significant concern [34,35,36]. In elderly patients, where the regenerative potential of bone tissue is inherently diminished, autologous bone harvesting is often impractical or poses considerable risks [37]. This review examines the primary categories of bone substitutes used in orthopedic applications, specifically distinguishing natural and synthetic materials. Natural substitutes include autologous, homologous, and heterologous grafts, while synthetic substitutes can be classified as biodegradable or non-biodegradable materials. Biodegradable options include bioactive ceramics (BC) [38], bioactive glass (BG) [39,40] and biodegradable polymers such as chitosan [41], polylactic acid (PLA) [42], polycaprolactone (PCL) [43] and collagen [44]. These materials are essential in bone regeneration, offering bioactive properties that promote osteointegration and cellular differentiation. Non-biodegradable materials, such as carbon nanotubes (CNT) [45,46], represent a frontier innovation in orthopedic biomaterials. Their exceptional mechanical properties and nanostructured surfaces facilitate osteogenic cell proliferation and differentiation while maintaining high biocompatibility and bone affinity [47,48].

## 2. Bone Grafts

Bone grafting is a widely employed technique in orthopedic surgery to facilitate bone repair, particularly in conditions such as nonunion, malunion, and delayed union. Bone grafts can be classified based on their origin, being either autologous (harvested from the same individual), homologous (obtained from a donor of the same species), or heterologous (sourced from different species, such as bovine or porcine). Additionally, bone grafts can be categorized based on their physicochemical properties, which include osteoconduction, osteoinduction, osteogenesis, osseointegration, and structural support—each playing a distinct role in the bone healing process. Osteoconduction refers to the ability of a biomaterial or graft to act as a scaffold that supports the growth of new bone tissue. Osteoconductive materials facilitate osteoblasts’ attachment, proliferation, and differentiation, promoting bone formation in the desired site [49,50]. This property is critical in filling bone defects or integrating implants such as dental or orthopedic prostheses [51]. Common osteoconductive materials include autografts, allografts, xenografts, calcium phosphates (e.g., hydroxyapatite and tricalcium phosphate), and bioactive glasses [52,53,54]. Osteoinduction is the process by which specific biomolecules or materials stimulate mesenchymal stem cells (MSCs) or precursor cells to differentiate into osteoblasts, thereby initiating new bone formation [55]. Bone morphogenetic proteins (BMPs) are among the most potent osteoinductive molecules, and they play a critical role in skeletal development and fracture healing. These biomolecules, whether naturally derived or synthetically produced, are extensively utilized in orthopedic and regenerative medicine to promote bone regeneration in cases of great defects or complex fractures [56]. Osteogenesis is the biological process by which specialized cells synthesize new bone tissue, primarily osteoblasts. It is fundamental for bone growth, repair, and maintenance. During osteogenesis, osteoblasts secrete collagen and non-collagenous proteins, which serve as a structural matrix for mineral deposition. Minerals such as calcium and phosphate then crystallize within this matrix, forming rigid, mechanically resilient bone tissue. Osteogenesis is a continuous process influenced by various hormonal, cytokine, and mechanical stimuli that regulate the dynamic balance between osteoblast-mediated bone formation and osteoclast-mediated resorption. Osseointegration describes the direct, structural, and functional connection between living bone and the surface of an implant without the presence of interposed soft tissue. The speed and success of osseointegration depend on the implant’s surface properties, including topography, chemical composition, and porosity, which influence osteoblast adhesion and proliferation. Several studies suggest that an implant does not necessarily require direct bone contact to osseointegrate, as factors such as primary stability and blood clot formation at the implant site play crucial roles in this process [57]. Among the most commonly used osseointegrative biomaterials are titanium implants, which can be either pure or coated with calcium phosphate to enhance bone-implant bonding [58,59,60]. These materials have demonstrated strong chemical stability, fostering robust integration with surrounding bone tissue. Structural support is another critical property of bone grafts and biomaterials, ensuring the mechanical integrity of the graft while promoting biological integration. The structural design of a graft—its porosity, mechanical strength, and degradation kinetics—must closely mimic that of native bone tissue to facilitate osteoconduction and osteogenesis. A well-structured graft provides a three-dimensional matrix with a favorable environment for bone cell attachment, proliferation, and differentiation. Structural support is particularly crucial in orthopedic applications, where the stability of bone grafts contributes to the safe and the integration of the graft materials with the surrounding bone [61,62]. Through a comprehensive understanding of these key properties, clinicians can optimize bone graft selection based on the specific needs of each clinical case, enhancing bone healing outcomes while minimizing complications. Future research in orthopedic biomaterials continues to explore novel scaffold designs, bioactive coatings, and advanced biotechnological approaches to further refine the efficacy and safety of bone graft substitutes in regenerative medicine.

### 2.1. Natural Grafts

**Figure 1:**
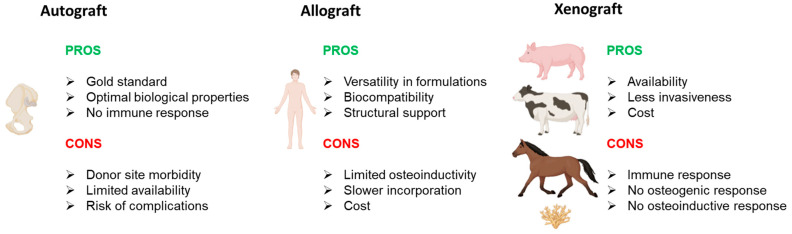
Pros and cons of natural bone grafts in use (from **left** to **right**): autograft, allograft, and xenograft. Images created with BioRender.com.

Natural bone grafts play a fundamental role in orthopedic regenerative medicine, offering a biological solution for bone replacement, regeneration, and repair. These grafts possess inherent properties that promote successful integration with the recipient’s bone, leveraging osteoconduction, osteogenesis, and osteoinduction mechanisms. One of their primary advantages is biocompatibility, as they originate from biological tissues and are generally well-tolerated by the human body, minimizing the risk of rejection. Additionally, their three-dimensional structure provides a natural scaffold for new bone formation, supporting the stability and regeneration of bone defects. However, natural bone grafts also present limitations, such as biological variability, which may lead to inconsistent clinical outcomes due to differences in the source material’s properties (Figure 1).

#### 2.1.1. Autograft

Autologous bone grafts—harvested from the same individual receiving the transplant—are considered the gold standard due to their high osteogenic potential among natural grafts. This is primarily attributed to their rich presence of growth factors and mesenchymal stem cells (MSCs) within the marrow portion and their excellent osteoinductive, osteoconductive, and osseointegration properties [63,64]. A key advantage of autologous grafts is the absence of immunological rejection, as the graft is recognized as “self” by the immune system. Additionally, their use is cost-effective, requiring only surgical and therapeutic materials for harvesting. Autologous bone grafts can be categorized based on the donor site. Intraoral sites include symphysis, mandibular ramus, maxillary tuberosity, edentulous ridges, zygomatic process, nasal spine, and exostoses, whilst extraoral sites include iliac crest, calvaria, and tibia [65,66]. Two primary types of autologous grafts are commonly used in orthopedic surgery, particularly in nonunion cases: cancellous and cortical bone grafts. Among these, cancellous bone grafts are the most widely utilized due to their high cellular concentration of osteoblasts and osteocytes, granting them superior osteogenic potential compared to other graft types [49]. The bone regeneration process following cancellous grafting occurs in distinct phases. Initially, the formation of new bone tissue occurs via a resorption–replacement mechanism. A local hematoma attracts mesenchymal stem cells (MSCs), which are critical for regeneration, and macrophages, which clear necrotic tissue. Subsequently, osteoblasts migrate to the periphery of the pre-existing trabecular structure, producing osteoid, which later undergoes mineralization, forming new bone. The process takes approximately 6 to 12 months to complete [67]. However, cancellous bone grafts have limited mechanical strength, making them unsuitable for weight-bearing applications.

In contrast, cortical bone grafts provide greater structural integrity [68]. The incorporation of cortical bone grafts follows a process known as creeping substitution, whereby the graft undergoes gradual resorption and simultaneous new bone deposition [49,69,70]. This process is predominantly mediated by osteoclasts, making cortical grafts slower to integrate than cancellous grafts [70]. Despite their biological advantages, autologous grafts pose several challenges. The harvesting procedure prolongs surgical time and is often associated with postoperative pain and aesthetic defects. Moreover, certain patient populations—such as the elderly, pediatric patients, and individuals with malignancies—may not be suitable candidates for autologous bone harvesting due to poor regenerative capacity or the risk of additional complications [71,72]. Furthermore, autologous bone grafts may fail if cellular elements within the graft do not survive transplantation. Additionally, complications such as infection, hematoma, excessive blood loss, nerve damage, hernia formation, vascular injuries, fractures, and chronic donor site discomfort have been reported, with incidence rates ranging between 8.5% and 20% [73,74,75]. These factors highlight the need for alternative strategies, such as synthetic bone substitutes and tissue-engineered scaffolds, to overcome the limitations associated with autologous bone grafting while maintaining optimal regenerative potential.

#### 2.1.2. Allograft

Homologous bone represents a viable alternative to autologous bone, as it is harvested from individuals of the same species as the recipient. Bone transplantation can be performed using living donors, such as femoral heads excised during hip replacement surgery, or from cadaveric donors, with the harvested material subsequently preserved in bone banks following appropriate processing [76,77,78]. Allografts are commonly available in multiple formulations, including powders, pastes, fibers, and structural blocks, each tailored for specific clinical applications [79]. These materials play a fundamental role in bone regeneration and defect repair, particularly when considering their biocompatibility, osteoconductivity, and mechanical properties. Powders and pastes are widely used for filling irregular bone defects due to their fine particle size, which ensures efficient adaptation to the defect morphology while enhancing osteoconductivity. Their ease of handling and ability to conform to complex anatomical structures make them particularly suitable for minimally invasive procedures and orthopedic applications [80]. The demineralized bone matrix (DBM), a widely utilized allograft material, belongs to this category, as it retains the organic matrix and growth factors essential for osteoinduction, while providing a scaffold for new bone formation [81,82]. However, the effectiveness of DBM remains controversial due to variability in BMPs content across different lots and the absence of standardized processing protocols. Despite its bioactivity, DBM lacks mechanical strength, requiring the use of carriers such as calcium sulphate or collagen to enhance its handling properties [83]. Fibers, on the other hand, offer improved mechanical interconnectivity and stability, promoting sustained cellular infiltration and vascularization during bone regeneration. The increased surface area provided by fibrous structures enhances osteointegration and accelerates new bone formation by facilitating cell migration and proliferation. Studies suggest that fiber-based allografts support vascularization more effectively than particulate forms, as their interconnected porous architecture allows for more efficient nutrient diffusion and vascular ingrowth, critical factors in successful bone healing [84]. Structural blocks, while less adaptable to irregular defects, are particularly valuable in load-bearing applications where mechanical stability is a priority. These blocks can serve as structural grafts in spinal fusion, large bone defect reconstruction, and orthopedic trauma management. Their composition and porosity can be optimized to balance strength and biological integration, promoting long-term graft incorporation and remodeling [33]. A key challenge in allograft application remains the balance between resorption and new bone formation. While these materials offer structural support, their limited osteoinductive properties necessitate the incorporation of bioactive factors or cellular components to enhance bone healing. The absorption capabilities of allografts vary depending on their processing technique. Indeed, freeze-dried and irradiated allografts offer extended shelf life and sterility, but their bioactivity may be compromised due to structural modifications during processing [63,70]. Gamma irradiation, commonly employed for sterilization, can degrade collagen fibers and reduce the osteoinductive potential of allografts [85,86]. Moreover, concerns about residual infective risks [87,88,89], donor availability, and the costs associated with biobanking remain significant limitations [90,91]. While allografts serve as a viable alternative to autografts in clinical practice, their clinical performance depends on factors such as graft preparation methods and host response. Current research is focused on optimizing porosity, improving integration with host tissue, and developing hybrid grafts that combine the advantages of multiple formulations to achieve superior clinical outcomes. Recent advances in tissue engineering aim to enhance allograft bioactivity through biomimetic modifications, such as ion substitution in hydroxyapatite or controlled delivery of growth factors [92,93]. These approaches seek to mitigate the limitations of traditional allografts, improving osteointegration and overall clinical outcomes.

#### 2.1.3. Xenograft

Heterologous bone grafts, derived from species different from that of the recipient, are primarily sourced from bovine or porcine origins [35,94]. Given their non-human derivation, these grafts present immunogenic challenges, necessitating extensive processing techniques that systematically eliminate proteins through chemical treatments or thermal processing. Coral-derived substitutes represent an emerging category of biomaterials with remarkable osteoconductive properties. These materials, predominantly composed of calcium carbonate, undergo hydrothermal conversion into calcium phosphate-based scaffolds, closely mimicking the trabecular architecture of natural bone. Their interconnected porous structure enhances cell adhesion and vascularization, promoting bone regeneration. Clinical studies have demonstrated their efficacy in facilitating new bone formation, particularly in load-bearing applications, where their compressive strength and osteoconductive potential make them a viable alternative to traditional grafting materials [95,96]. Likewise, equine xenografts, offer significant advantages over traditional bovine sources. Unlike bovine-derived substitutes, equine xenografts undergo enzymatic deantigenation processes that preserve the bone’s collagen structure, thereby enhancing osteointegration and long-term stability [97]. Studies have demonstrated that equine-derived xenografts effectively support osteogenic differentiation when combined with stem cells, showing increased collagen type I expression and calcium deposition. These findings suggest their superior potential for periodontal and maxillofacial bone regeneration [98]. Compared to conventional bone grafts, both coral-derived substitutes and equine xenografts exhibit high biocompatibility and low immunogenicity, reducing the risk of adverse reactions while maintaining structural integrity [99]. As research advances in bone tissue engineering, these biomaterials continue to gain recognition as promising alternatives for enhancing bone repair and regeneration in clinical practice. Despite their immunological limitations, heterologous grafts offer several advantages over autologous bone, including the abundant availability of material, rendering them suitable for large defects, as well as the reduced invasiveness of the procedure, as patients do not require additional surgical interventions for bone tissue harvesting. Accordingly, shorter healing times are necessary and a lower risk of postoperative morbidity [100]. Furthermore, heterologous bone substitutes exhibit favorable mechanical properties, good osteoconductivity, and cost-effectiveness [101,102]. However, several disadvantages persist, including the potential for infection at the implantation site, potential premature graft resorption before complete replacement by newly formed bone, and the risk of pathogen transmission or immune sensitization to donor proteins [103].

### 2.2. Future Perspectives on Natural Grafts

Recent advancements in regenerative engineering and tissue engineering have introduced novel methodologies to enhance the efficacy of natural grafts, including autografts, allografts, and xenografts. These approaches incorporate growth factors, bioengineered scaffolds, advanced sterilization processes, and cellular therapies to improve osteointegration, bone regeneration, and long-term clinical outcomes. Autologous bone grafts remain the gold standard in bone reconstruction due to their inherent osteogenic, osteoinductive, and osteoconductive properties. Recent studies have shown that pre-treating autologous grafts with growth factors, such as bone morphogenetic proteins (BMPs), platelet-derived growth factors (PDGF), and vascular endothelial growth factor (VEGF), can significantly enhance bone regeneration while minimizing post-graft resorption [104,105]. Furthermore, mesenchymal stem cells (MSCs) derived from the patient’s own bone marrow or adipose tissue have been integrated into grafts to further accelerate osteogenesis [106,107]. Another innovative approach is the use of 3D bioprinting to fabricate personalized implants from autologous bone cells combined with biomimetic scaffolds, which are currently undergoing clinical trials [108]. These patient-specific constructs enhance mechanical stability, promote vascularization, and reduce the risks associated with traditional grafting techniques. Allogeneic bone grafts, although widely used, face challenges related to immune rejection and limited osteoinductive potential. To address these limitations, researchers have developed decellularization techniques combined with stem cell revitalization [109]. This method involves removing antigenic cellular components from donor tissue while preserving the extracellular matrix (ECM) to maintain osteoconductive properties [110]. Stem cell revitalization involves seeding the decellularized allograft with patient-derived stem cells (e.g., MSCs or induced pluripotent stem cells, iPSCs), which improves osteogenic potential and graft integration [111]. In addition, studies have shown that pre-conditioning allografts with bioactive molecules such as BMP-2 and transforming growth factor-β (TGF-β) can further enhance osteoinduction [112,113]. Another breakthrough is the application of vascularized allografts, where prevascularization techniques using endothelial cells or angiogenic growth factors enable faster vascular integration and bone remodeling, reducing failure rates [114,115,116]. Xenogeneic bone grafts offer an alternative to human grafts. However, concerns about immunogenicity and disease transmission have historically limited their clinical application. Recent advancements in advanced sterilization processes—such as gamma radiation combined with enzymatic treatments—have been shown to better preserve osteoinductive properties compared to traditional heat or chemical treatments [117,118,119]. Additionally, deproteinization and collagen crosslinking techniques have been employed to enhance biocompatibility and reduce immunogenic responses [120,121]. Bioengineered xenografts coated with growth factors and stem cells are also being investigated to mimic natural bone remodeling processes more effectively. As tissue engineering continues to evolve, the combination of biomaterials, gene therapy, and regenerative medicine will likely revolutionize the field of bone grafting. Future research aims to optimize the integration of bioprinted scaffolds with living cells, enhance graft vascularization, and explore gene-editing technologies (e.g., CRISPR-Cas9) to improve the osteoinductive properties of grafts [122,123,124,125]. By leveraging these innovations, natural grafts will become more personalized, durable, and effective, offering improved clinical outcomes for patients with large bone defects, degenerative bone diseases, and orthopedic trauma.

### 2.3. Market Availability

The global market for bone graft substitutes is vast, featuring a variety of advanced formulations from well-established brands. Among the commercially available options, allograft-based products such as Grafton^®^ (demineralized bone matrix-based, DBM), OsteoSelect^®^ DBM Putty, and Opteform^®^ (a combination of allograft and carriers to enhance handling properties) are frequently utilized in clinical settings. These products capitalize on the regenerative potential of human bone-derived materials, with Grafton^®^ and OsteoSelect^®^ being particularly favored for their osteoconductive and osteoinductive properties. Xenografts, including Bio-Oss^®^ (bovine-derived) and Bioteck Bio-Gen^®^ (equine-derived), offer distinct advantages in both maxillofacial and orthopedic reconstructions. These materials are prized for their structural and compositional similarities to human bone, making them suitable for enhancing bone regeneration. Studies have shown that bovine-derived xenografts, such as Bio-Oss Collagen, are particularly effective in periodontal regeneration and bone augmentation, with significant clinical improvements observed in furcation defects and alveolar ridge augmentation [126]. These xenografts provide a scaffold for new bone growth, facilitating the regeneration of lost tissues in cases of trauma, infection, or oncologic resections [127]. In terms of osteogenic potential, Bio-Oss^®^ and Bioteck Bio-Gen^®^ are frequently combined with growth factors or barrier membranes to enhance their effectiveness. For instance, Bio-Oss Collagen paired with Bio-Gide (a collagen membrane) has been shown to yield favorable outcomes in periodontal regeneration, promoting bone formation while preventing epithelial downgrowth. Similarly, the addition of fibrin sealing systems like TISSEEL has shown promise in enhancing the regenerative capacity of xenograft materials [128]. The success of these bone graft substitutes depends heavily on their osteoconductive properties, which allow the graft to serve as a scaffold for host bone cells to attach and proliferate. While autografts remain the gold standard due to their osteogenic, osteoinductive, and osteoconductive properties, allografts and xenografts offer viable alternatives, particularly in cases where autogenous bone is insufficient or unavailable [129].

## 3. Tissue Scaffold

Bone tissue is primarily composed of hydroxyapatite as its mineral phase. However, several impurity ions are present within the hydroxyapatite lattice, including carbonate, magnesium, and sodium ions. Carbonate, in particular, is abundant, accounting for approximately 4–8% of the mineral content. As a result, bone and other hard tissues can be characterized as carbonate-substituted hydroxyapatite (C-HA) [130,131,132]. Synthetic bone substitutes, classified as alloplastic biomaterials, are widely recognized for their high osteoconductivity and are suitable for a broad range of grafting applications. These materials do not elicit immune reactions, enable shorter recovery times, exhibit no systemic or local toxicity, are easily sterilizable, and are commercially available [68]. They are produced in various formulations, including powders, putties, pellets, and implant coatings [66] and they can also serve as carriers for therapeutic agents such as antibiotics, making them particularly advantageous for treating bone defects associated with infections [133,134,135]. While some calcium phosphate cement (CPCs) exist as pure, single-compound formulations, most commercially available products incorporate mixed calcium salts in varying concentrations [136]. These materials possess several advantageous properties, including slow biodegradation, high compressive strength, and exceptional osteointegration, allowing for direct interdigitation of the host bone with the rough crystalline graft interface [137,138,139]. However, the in vivo mechanical strength of calcium phosphate substitutes remains inferior to that of normal cancellous bone [34], primarily due to their inherent brittleness under tensile or shear forces, which frequently accompany compressive loads in physiological conditions [35,136]. To address these mechanical shortcomings, researchers have explored the incorporation of magnesium-based biomaterials due to their favorable mechanical properties and biocompatibility. Among these innovations, magnesia screws, composed primarily of magnesium oxide (MgO), have emerged as promising bioresorbable fixation devices for orthopedic applications [140,141]. Bone substitutes currently available on the market are categorized into three main groups: ceramics, bioactive glasses, and polymers.

### 3.1. Ceramics

Ceramics are solid materials composed of metal or non-metal compounds that undergo a shaping process followed by thermal treatment at elevated temperatures to achieve their final structure [142]. Their application in orthopedic and dental surgery has been well established for over three decades, following diffraction and chemical analyses that demonstrated the predominant presence of calcium hydroxyapatite (HA) in the inorganic phase of bone tissue, accounting for approximately 70% of its composition. This finding suggested that HA could serve as an ideal biomaterial for facilitating bone regeneration by closely mimicking the native mineral phase [39,143]. Chemically, HA is an apatite mineral containing a hydroxyl group and is primarily composed of calcium cations and phosphate anions in a Ca^2+^/PO_4_^3−^ ratio of 1.67 (Ca_5_(PO_4_)_3_OH) [144]. Consequently, HA is regarded as the archetype of calcium phosphate ceramics (CaPs), a widely studied class of biomaterials. In addition to HA, tricalcium phosphate (TCP), the amorphous phase of HA, represents another essential CaP utilized in bone tissue engineering, either in its pure form or as a biphasic composition combined with HA (BCP). Hydroxyapatite (HA) is characterized by remarkable mechanical strength. It remains in the body for extended periods, whereas the more porous TCP, particularly in its β-phase, undergoes biodegradation within six weeks of implantation in the bone formation site. While HA exhibits high crystallinity and resists in vivo degradation, TCP is resorbable and highly soluble TCP exists in two distinct crystal phases (alpha and beta), sharing similar chemical composition but differing in crystallographic features that influence their resorption behavior [145,146]. A biphasic calcium phosphate formulation comprising 40–60% TCP and 60–40% HA provides an optimized balance between mechanical support and bone resorption dynamics [147]. HA-TCP ceramics are well established, demonstrating excellent safety and efficacy in bone replacement procedures. These materials are commercially available in multiple formats, including blocks, granules, and injectable formulations. Several studies show spherical HA particles enhance osteointegration and mitigate inflammatory responses [148,149]. Their biocompatibility and compositional similarity to natural bone contribute to their exceptional osteoconductive and osteointegrative properties [150]. Several parameters influence HA-based ceramics’ biological performance, including chemical composition, phase transformation, microstructure, pore size, and porosity. Depending on the specific functional requirements and implantation site, both porous and dense bioceramics are utilized in surgical applications. Experimentally, porous ceramics exhibit reduced mechanical strength, yet their enhanced porosity is advantageous for applications requiring drug delivery or implantation in low-load-bearing regions, such as maxillofacial reconstruction [151,152,153]. Successful osteointegration is contingent upon implant porosity, particularly its size, volume, and interconnectivity. Studies have demonstrated that bone ingrowth necessitates a minimum pore size of approximately 100–135 µm, with increased interconnectivity facilitating enhanced bone regeneration, particularly in fixation procedures [154]. Additionally, ceramics serve as foundational materials for composite formulations. Current research focuses on the synthesis of HA-matrix composites incorporating fine particles, micro-lamellae, or fibers to improve their mechanical strength and toughness, rendering them suitable for load-bearing applications in hard tissue replacement [155,156]. While ceramics have traditionally played a fundamental role in load-bearing applications due to their bioactivity and osteoconductive properties, their inherent brittleness necessitates the exploration of alternative materials with improved mechanical performance. In this regard, magnesium-based orthopedic screws represent a promising solution, offering a degradable and bioactive alternative to conventional metallic implants. Magnesium screws gradually degrade in vivo, simultaneously releasing bioactive magnesium ions that promote osteogenesis while preventing stress shielding [157,158]. Compared to traditional metallic implants, which may cause long-term complications such as stress-induced bone resorption, magnesium-based devices offer a more biocompatible and resorbable alternative [159,160]. Recent studies have highlighted the importance of optimizing the geometric design of magnesium screws to enhance their mechanical integrity and corrosion resistance. Stress-induced corrosion is a critical challenge in magnesium-based orthopedic devices, as it can lead to premature implant degradation and compromise fracture fixation. Research has demonstrated that thread geometry, pitch, and width significantly influence stress distribution, thereby affecting the degradation rate of the implant. Finite element analysis (FEA) has been employed to optimize the screw design, revealing that triangular thread types with specific pitch and width parameters can effectively reduce stress concentration and improve corrosion resistance [161]. In vivo studies using animal models have shown that optimized magnesium screws maintain sufficient mechanical stability throughout the bone healing process, promoting new bone formation while minimizing fibrous encapsulation [161,162]. Furthermore, clinical evaluations indicate that magnesium screws exhibit favorable biocompatibility, with no significant inflammatory response or adverse gas accumulation [163,164]. The controlled degradation of these implants ensures gradual load transfer to the healing bone, enhancing integration and reducing the risk of delayed union or nonunion.

### 3.2. Bioactive Glasses

**Figure 2:**
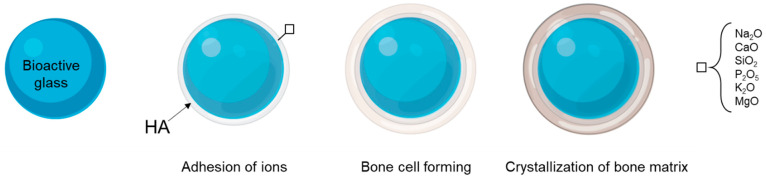
Bioactive glass development model (from left to right): adhesion ions to surface forming bone hydroxyapatite-like; the bone cell forming on the surface of the coated bioactive glass; complete crystallization of the bone matrix.

Bioactive glasses, a silicate-based material class, exhibit osteoconductive and osteoinductive properties when formulated with specific compositions. By modulating the relative proportions of sodium oxide (Na_2_O), calcium oxide (CaO), and silicon dioxide (SiO_2_), various bioactive glass formulations can be synthesized to optimize their regenerative potential [165,166]. These materials are mainly distinguished by their capacity to establish strong interfacial bonds with bone and soft tissues [167,168]. Upon implantation, bioactive glasses initiate the formation of a dense hydroxyapatite carbonate layer on their surface, mimicking the mineral phase of bone and thereby enhancing cellular adhesion (Figure 2) [169]. Increasing evidence suggests that the regenerative capacity of bioactive glass scaffolds is determined by factors such as composition, microstructural properties, and fabrication techniques, frequently demonstrating superior performance compared to ceramics [170,171]. Based on their compositional attributes and specific physicochemical characteristics, bioactive glasses are classified into 45S5, 58S, and 1393 groups [150,172].

### 3.3. Polymers

**Figure 3:**
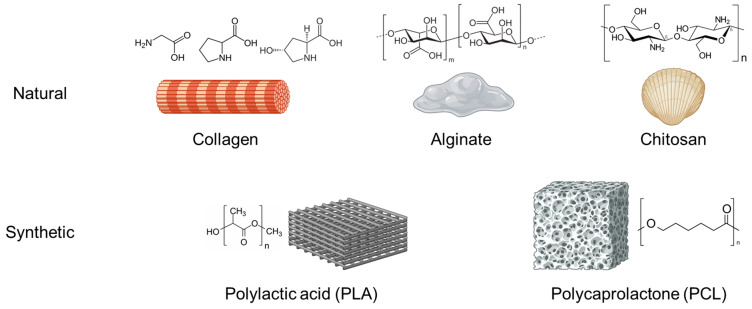
Diagram of the natural (collagen, alginate, and chitosan) and synthetic (PLA, PCL) polymers in use. Image created with BioRender.com.

Polymers employed in orthopedic regenerative medicine are categorized as natural or synthetic. Natural polymers include biocompatible and biodegradable substances such as collagen, chitosan, and alginate, which are highly suitable for medical applications (Figure 3). Collagen, the principal component of connective tissue, is extensively utilized in scaffolds to support cellular adhesion and proliferation, thereby facilitating the regeneration of bone and cartilage tissues. In contrast, synthetic polymers such as polylactic acid (PLA) and polycaprolactone (PCL) offer distinct advantages, including tunable mechanical properties and precisely controllable degradation rates. These features enable their use in applications where customized material properties are essential. Additionally, carbon nanotubes have emerged as a promising innovation in orthopedic tissue engineering. This is due to their exceptional mechanical and electrical characteristics and their capacity for chemical functionalization, which further enhances their bioactivity and integration potential.

#### 3.3.1. Chitosan

Chitosan is a natural polymer derived from chitin, a widely abundant biopolymer found in the cell walls of fungi and the exoskeleton of crustaceans such as crabs and shrimp [173,174]. Chemically, chitosan is a polysaccharide composed of D-glucosamine and N-acetyl-D-glucosamine units linked by β(1→4) glycosidic bonds [175]. The transformation of chitin into chitosan occurs through a deacetylation process, wherein the removal of acetyl groups enhances its solubility in weak acids and imparts distinctive properties that render it highly suitable for biomedical applications [176]. One of the primary advantages of chitosan is its ability to form gels and films, which allows it to be processed into diverse formats, including sponges, membranes, and hydrogels, thereby making it an ideal scaffold material for bone and cartilage regeneration [177]. Chitosan scaffolds can undergo modifications to improve their mechanical strength and biological functionality. For instance, the incorporation of other biopolymers, such as collagen or hyaluronic acid, or the reinforcement with bioceramic nanoparticles like hydroxyapatite, significantly enhances their structural integrity, effectively mimicking the extracellular matrix of native bone tissue [178]. Chitosan’s polymeric structure contains free amino groups that enable facile chemical modifications, allowing for the conjugation of bioactive molecules such as growth factors, pharmaceuticals, or peptides, further broadening its applicability in regenerative medicine [174]. In the context of orthopedic applications, chitosan plays a pivotal role in promoting cartilage regeneration [179]. Since articular cartilage is an avascular tissue with limited intrinsic regenerative capacity, chitosan-based scaffolds offer structural support to chondrocytes, facilitating cartilage matrix production and enhancing tissue repair [180]. Furthermore, due to its hydrogel-forming properties, chitosan provides a hydrated microenvironment conducive to cell proliferation and differentiation. A significant attribute of chitosan is its biodegradability; upon implantation, it gradually decomposes into non-toxic degradation products, either absorbed or excreted by the body. The degradation rate can be controlled by altering the degree of deacetylation and molecular weight, allowing for the design of scaffolds that degrade in synchronization with new tissue formation [181]. Ongoing research on chitosan-based scaffolds has demonstrated their potential in clinical applications, with a tricalcium phosphate/chitosan scaffold currently undergoing phase III clinical trials (ClinicalTrials.gov Identifier: NCT02081885) for the treatment of mandibular fractures [182].

#### 3.3.2. Collagen

Collagen is one of the most abundant structural proteins in the human body, forming a fundamental component of the extracellular matrix across various tissues, including bone, cartilage, skin, and tendons, accounting for approximately 20–30% of total body proteins in mammals [183]. Similar to chitosan, collagen has garnered significant attention in regenerative medicine due to its exceptional biocompatibility, biodegradability, and ability to support cellular growth [184]. Structurally, collagen proteins are characterized by a unique triple-helix configuration, composed of three pro-collagen polypeptide chains—two α1 and one α2 chains—predominantly rich in glycine (Gly), proline (Pro), and 4-hydroxyproline (Hyp), forming a repeating Gly-Pro-Hyp triplet [185]. This distinctive structure confers remarkable tensile strength and thermal stability. Among the various collagen types, types I, II, and III are most commonly employed in regenerative medicine, each exhibiting specific properties tailored for specific biomedical applications [186]. Type I collagen is the most prevalent, constituting over 90% of the organic matrix of bone and serving as a principal protein component in numerous tissues, including tendons, ligaments, cornea, cartilage, pancreas, and skin [187]. The assembly of collagen fibers into fibrils is strongly influenced by environmental parameters such as pH, ionic strength, and temperature, which must be precisely controlled in in vitro fibrillogenesis [188].

Collagen molecules possess distinct cell-adhesion domains, facilitating cellular interactions essential for tissue regeneration. These domains interact with specific receptors on the cell surface, promoting adhesion, proliferation, and differentiation [189]. In bone regeneration, osteogenic cells adhere to collagen scaffolds, initiating bone matrix deposition, whereas in cartilage regeneration, chondrocytes proliferate and secrete extracellular matrix components essential for cartilage formation [166,190,191]. Type II collagen plays a significant role in cartilage regeneration by forming a specialized matrix supporting chondrocyte proliferation and enhancing cartilage-specific extracellular matrix components [192]. Another advantage of is its ability to form injectable in situ gels. Collagen-based hydrogels can be delivered directly into tissue defects, where they undergo gelation, creating a supportive matrix for cellular infiltration and tissue regeneration. This minimally invasive approach is especially valuable in orthopedic applications, where precision and reduced surgical trauma are critical considerations [193,194]. The incorporation of bioactive agents into collagen scaffolds has also been investigated, with studies demonstrating that dexamethasone-loaded collagen scaffolds promote the osteogenic differentiation of mesenchymal stem cells (MSCs) in vitro and enhance ectopic bone formation following subcutaneous implantation in animal models [195]. Several clinical studies have employed porous collagen scaffolds, demonstrating promising outcomes in bone regeneration. However, certain complications have been reported, including inflammation, hematoma formation, and other adverse effects [196,197,198]. In addition to its use as a differentiation-promoting biomaterial, collagen scaffolds can be seeded with MSCs, enabling their differentiation into osteoblasts or chondrocytes, thereby facilitating bone and cartilage regeneration. This combined approach represents a highly promising therapeutic strategy for the treatment of extensive bone and cartilage defects [199].

#### 3.3.3. Alginate

Alginate, a natural polysaccharide predominantly derived from brown algae, consists of mannuronic acid (M) and guluronic acid (G) residues arranged in variable sequences [200]. Its chemical composition can be tailored, influencing its physicochemical and mechanical properties. Alginate is particularly attractive for regenerative medicine applications due to its outstanding biocompatibility, its ability to form hydrogels under mild physiological conditions, and its ease of processing into various forms, including hydrogels, microcapsules, and porous sponges [201,202,203]. In orthopedic tissue engineering, alginate is primarily employed in hydrogel form. Alginate hydrogels are synthesized via an ionic gelation mechanism, wherein solubilized alginate undergoes crosslinking upon exposure to divalent cations such as calcium (Ca^2+^). This gelation process occurs under ambient conditions, preserving the viability and biological activity of encapsulated cells or bioactive molecules, thereby making alginate an ideal biomaterial for tissue engineering applications [204,205,206]. One of the key advantages of alginate in tissue regeneration is its ability to provide a hydrated, three-dimensional microenvironment that facilitates cell proliferation and differentiation [207]. Alginate hydrogels mimic the extracellular matrix, providing structural support and promoting cellular interactions. In cartilage tissue engineering, alginate-based scaffolds enable the encapsulation of chondrocytes, maintaining their phenotype and stimulating the production of type II collagen and proteoglycans. Furthermore, the porous architecture of alginate hydrogels ensures efficient diffusion of nutrients and metabolic byproducts, supporting long-term cell viability and function [208,209].

#### 3.3.4. Polylactic Acid (PLA)

Polylactic acid (PLA) is a biodegradable and biocompatible synthetic polymer derived from lactic acid, which is obtained through the fermentation of renewable resources such as corn starch or sugars [210,211]. Structurally, PLA is a linear polyester composed of repeating units of lactic acid, which can be arranged in different stereochemical configurations, including L-PLA (poly-L-lactic acid), D-PLA (poly-D-lactic acid), or a combination of both, such as PLLA (poly-L-lactic acid) and PDLLA (poly-D, L-lactic acid) [212,213,214]. The stereochemical arrangement significantly influences the polymer’s physical and mechanical properties, including melting temperature, crystallinity, and degradation rate. For instance, PLLA is more crystalline and degrades more slowly than PDLLA, which is amorphous and exhibits a faster degradation profile [215,216]. One of the primary challenges in utilizing PLA for bone regeneration lies in its inherent brittleness and relatively low tensile strength compared to native bone. To address these limitations, PLA can be reinforced with other materials, such as bioactive ceramics (e.g., hydroxyapatite, β-tricalcium phosphate) or other biodegradable polymers (e.g., poly(glycolic acid)—PGA), to enhance its mechanical performance and bioactivity [217,218]. These composite materials provide improved structural integrity and promote bone mineralization and the formation of new bone tissue. PLA is also widely employed in the fabrication of orthopedic fixation devices, including screws, plates, and pins, which serve to stabilize fractures or facilitate joint reconstruction [219]. These devices offer temporary mechanical support and gradually degrade within the body, eliminating the necessity for a secondary surgical procedure for removal. PLA degradation occurs via hydrolysis of ester bonds, producing lactic acid, which is subsequently metabolized into water and carbon dioxide [220,221].

#### 3.3.5. Polycaprolactone (PCL)

Polycaprolactone (PCL) is a semi-crystalline polyester characterized by a low melting temperature (approximately 60 °C) and a slow degradation rate, properties that make it particularly advantageous for a variety of biomedical applications [222,223]. PCL is synthesized through the ring-opening polymerization of caprolactone, a cyclic monomer. Its molecular structure comprises repeating ε-caprolactone units linked by ester bonds [224]. This configuration imparts PCL with significant flexibility and tensile strength, which are critical properties for materials intended for tissue regeneration [225,226]. Within the field of bone regeneration, PCL is utilized to fabricate three-dimensional scaffolds that provide structural support for the growth and differentiation of osteogenic cells. These scaffolds can be differently produced using various processing techniques, including electrospinning, 3D printing, and particle leaching [227]. Among these, electrospinning enables the generation of ultrafine fibers that mimic the architecture of the natural extracellular matrix, thereby promoting cellular adhesion and proliferation [228,229]. A key advantage of PCL lies in its prolonged biodegradation profile. PCL degrades via the hydrolysis of ester bonds, yielding carboxylic acids and alcohols metabolized by the body. Due to its slow degradation rate, which can extend from several months to years, PCL maintains mechanical stability over extended periods, thereby providing sufficient time for tissue remodeling and forming a new extracellular matrix [223,230]. In addition to its applications in orthopedic regeneration, PCL is employed in other areas of regenerative medicine, including soft tissue engineering and wound healing. Its biocompatibility and versatility render it suitable for fabricating absorbable sutures, nerve regeneration matrices, and scaffolds for skin tissue repair [231,232].

#### 3.3.6. Carbon Nanotubes (CNT)

Carbon nanotubes (CNTs) are extensively employed as reinforcement agents to enhance the mechanical properties of polymeric scaffolds, improving the overall strength and rigidity of structures used for bone and cartilage tissue engineering [233,234]. Integrating CNTs with polymers produces nanocomposites that more closely replicate the mechanical characteristics of natural bone. A crucial consideration in the biomedical application of CNTs is their functionalization [235,236]. Pristine CNTs tend to be hydrophobic and prone to aggregation, which can limit their effectiveness. However, chemical functionalization can improve their dispersibility in polymer matrices, thereby enhancing their biocompatibility [237,238]. Functional groups can also be employed to anchor bioactive molecules, such as growth factors or peptides, further improving cellular interactions with CNT-based scaffolds. One of the most promising applications of CNTs in bone tissue engineering is their ability to enhance the electrical conductivity of scaffolds [239,240]. Studies have demonstrated that electrical stimulation can positively influence the proliferation and differentiation of osteogenic cells [241,242]. Due to their exceptional conductivity, CNTs can be incorporated into polymeric scaffolds to create electroactive platforms supporting bone cell growth and maturation [243,244,245,246,247]. Furthermore, CNTs offer potential advantages for drug-controlled release and growth factors. Their large specific surface area and functionalization capabilities make them ideal candidates for the targeted delivery of therapeutic molecules directly to the regeneration site. This targeted approach improves treatment efficacy while minimizing systemic side effects [248,249]. Beyond bone tissue engineering, CNTs are also being explored for their potential to enhance cartilage regeneration. Since cartilage is an avascular tissue with limited self-repair capabilities, CNTs incorporated into polymeric scaffolds can provide mechanical reinforcement while promoting chondrocyte proliferation and differentiation [250,251]. Preliminary studies have indicated that CNTs can modulate the expression of chondrogenic markers, thereby facilitating the development of high-quality cartilage tissue [252,253,254]. Despite these promising applications, concerns remain regarding the biocompatibility and potential toxicity of CNTs [46,255,256]. It is imperative that CNTs undergo rigorous purification and functionalization processes to mitigate the risks of inflammatory responses or cytotoxicity. Ongoing research continues to refine the application of CNTs in regenerative medicine to ensure their safety and efficacy for clinical use.

## 4. Conclusions

In conclusion, the extensive exploration of bone matrices in orthopedic applications, ranging from natural autografts and allografts to advanced synthetic alternatives such as ceramics, polymers, and nanotubes, underscores a rapidly evolving field characterized by continuous innovation and expanding therapeutic possibilities. Each type of bone matrix presents a distinct set of advantages and limitations, and the strategic selection of the most appropriate material plays a crucial role in determining clinical outcomes. Characterizing the physicochemical properties of these matrices and studying the biological applications are fundamental to advancing bone regeneration strategies and optimizing patient-specific treatment approaches. This review highlights the necessity of an informed and tailored approach in selecting bone matrices, emphasizing key parameters such as biocompatibility, osteoconductivity, osteoinductivity, regenerative potential, and potential risks. Furthermore, integrating natural and synthetic materials fosters the development of hybrid solutions that capitalize on the strengths of both categories, enhancing their overall efficacy. The synergy between biological scaffolds and engineered biomaterials paves the way for next-generation regenerative strategies, facilitating superior clinical outcomes and improved long-term functionality in orthopedic and maxillofacial reconstructions. By promoting ongoing research and technological advancements, this study underscores the transformative potential of bone matrices in contemporary orthopedic practice. Future investigations should focus on refining material properties, optimizing scaffold architectures, and incorporating bioactive molecules to enhance bone healing and integration further. The continuous evolution of biomaterials and tissue engineering approaches holds significant promise for addressing complex bone defects, improving patient recovery, and advancing the regenerative medicine field.

## Data Availability

Not applicable.
